# Phenomics in sport: Can emerging methodology drive advanced insights?

**DOI:** 10.3389/fnetp.2022.1060858

**Published:** 2022-11-24

**Authors:** Adam W. Kiefer, David T. Martin

**Affiliations:** ^1^ Department of Exercise and Sport Science, University of North Carolina at Chapel Hill, Chapel Hill, NC, United States; ^2^ Apeiron Life, Menlo Park, CA, United States; ^3^ School of Behavioral and Health Sciences, Australia Catholic University, Melbourne, NSW, Australia

**Keywords:** phenomics, phenotypic plasticity, hormesis, critical windows, phenotypic expression

## Abstract

Methodologies in applied sport science have predominantly driven a reductionist grounding to component-specific mechanisms to drive athlete training and care. While linear mechanistic approaches provide useful insights, they have impeded progress in the development of more complex network physiology models that consider the temporal and spatial interactions of multiple factors within and across systems and subsystems. For this, a more sophisticated approach is needed and the development of such a methodological framework can be considered a Sport Grand Challenge. Specifically, a transdisciplinary phenomics-based scientific and modeling framework has merit. Phenomics is a relatively new area in human precision medicine, but it is also a developed area of research in the plant and evolutionary biology sciences. The convergence of innovative precision medicine, portable non-destructive measurement technologies, and advancements in modeling complex human behavior are central for the integration of phenomics into sport science. The approach enables application of concepts such as phenotypic fitness, plasticity, dose-response dynamics, critical windows, and multi-dimensional network models of behavior. In addition, profiles are grounded in indices of change, and models consider the athlete’s performance or recovery trajectory as a function of their dynamic environment. This new framework is introduced across several example sport science domains for potential integration. Specific factors of emphasis are provided as potential candidate fitness variables and example profiles provide a generalizable modeling approach for precision training and care. Finally, considerations for the future are discussed, including scaling from individual athletes to teams and additional factors necessary for the successful implementation of phenomics.

## 1 Introduction

Maximizing an athlete’s availability and performance capabilities is central to success in competitive sports, with great time and effort invested to reduce injury, illness, and extreme fatigue. Despite decades of research targeting these topics, injury and re-injury rates do not appear to be decreasing (Conte et al., 2016; Mack et al., 2020; Westermann et al., 2016) and generalized health prediction remains elusive (Van Eetvelde et al., 2021). Applied sport science has predominantly utilized component-focused linear additive models of causality, with single-factor mechanisms emphasized with a limited account of environmental factors. For example, recent discoveries around biological markers supported the transition from genetics to (gen)-omics with the promise these new data would uncover high-resolution mechanistic underpinnings of physical capabilities (Ehlert et al., 2013), physical fitness trainability (Bouchard et al., 2011), soft tissue injury risk (Georgiades et al., 2017), concussion diagnosis and recovery (Gordon, 2010), and overall sport achievement (Ehlert et al., 2013). However, these discoveries have not been the promised panacea, as no reliable candidate genes or genome-wide associations have been discovered. Instead, inconsistent results have led to calls for expansion from epigenetics (Ehlert et al., 2013) into phenotypes with larger cohorts (Wang et al., 2016). Specific to physiology, there is a need to move beyond the molecular level toward a broader framework for how multiscale organ systems coordinate and integrate to promote emergent, adaptive behavior for successful performance (Balagué et al., 2022).

While much of what is known today is owed to component-focused linear modeling, it has unfortunately led to an impasse in understanding and predicting performance and health in sport. It is a barrier made up of underdetermined models that do not account for the complex interaction of integrated factors that drive athlete performance across both time and scale. Applied sport science is therefore in need of a more sophisticated approach to tackle what can be considered a *Sport Grand Challenge*—the precise profiling and modeling of complex interactions among the various components and sub-components of training and recovery to inform the successful risk management associated with athlete health. The purpose of this paper is to introduce a new framework that leverages emerging technology and methodologies, in combination with decades of work in evolutionary biology, to more accurately and comprehensively profile, model and predict athlete performance and health (Section 2). Several sport science domains are provided as example priorities for integration into this new approach, with specific factors of emphasis related to each (Section 3). We conclude with considerations for the future (Section 4).

## 2 Phenomics

Genomics-based precision medicine has shown great promise for treating specific disease states (Collins and Varmus, 2015; Ashley, 2016), with associated methodologies and modeling approaches foundational for solving our Sport Grand Challenge. However, nearly a decade ago, scientists realized that a genomics framework alone is insufficient, and advocated for innovative approaches for the detection, measurement and analysis of a range of biomedical data that move beyond traditional (gen)-omics data by taking into account behavioral, physiological and environmental parameters (Mirnezami et al., 2012; Collins and Varmus, 2015). These calls have since been expanded to prioritize high-resolution *phenomics* and environmental exposure data as a critical step for precision medicine to reach its full potential (Denny and Collins, 2021). It is with this focus that a new framework can emerge. The transdisciplinary study of phenomics captures the many dimensions of phenotypic change that arise from the interaction of an individual’s genetic make-up with environmental factors (Houle et al., 2010). As current phenomics approaches are based primarily on human medicine models, they risk repeating past mistakes of early human genome discussions on whether isolated components should be the focus (Lewin, 1986; Angier, 1990). A human medicine approach also relies on expensive, high-fidelity medical imaging and screenings conducted in relatively sterile laboratory settings that do not always facilitate practical and scalable implementation in sport.

Importantly, phenomics has also been a burgeoning area of research in the plant and evolutionary biology sciences (Shahzad et al., 2021), with established research lines in both lab-controlled and field environments (Lürig et al., 2021) borne out of comprehensive biological phenotyping and the introduction of the conceptual phenome in 1967 (Soule, 1967). These fields have standardized protocols and techniques that specialize in the comparatively low-cost, non-destructive screening of living organisms in their natural environment to index real-time multidimensional phenotypic change (West-Eberhard, 1989; Agrawal, 2001; Burggren, 2014; Hairmansis et al., 2014). This work is based on indexing change in biological *fitness,* defined as the rate of change (e.g., growth) of a genotype, phenotype or even a specific population of organisms relative to fluctuations in the environment (Laughlin et al., 2020). Thus, it is typically framed regarding organism survival: examinations of mortality rate, reproduction rate, and growth rate are examples of well-defined biological fitness variables for study (*cf.* Burggren and Mueller, 2015).

It is this approach to phenomics for which we advocate as foundational to solving the Sport Grand Challenge. Our proposed sport science framework is grounded in the phenotypic expression of the athlete’s performance in response to important and meaningful environmental influences. This expression is based on individual performance-relevant fitness variables. These variables must first be accurately identified, with each fitness variable part of a specific domain, or phenotype, that is then connected to other phenotypes. Thus, the approach moves beyond the traditional linear use of mechanism through the construction of a multidimensional phenomic profile (or network) across multiple domains. This makes the phenomic profile, and associated component phenotypes, adherent to dynamical explanatory (e.g., network) models that can predict the behavior of similar individuals (i.e., systems) and adhere to a *many-to-one* principal—i.e., outcomes realized through an array of potential mechanistic underpinnings, or varying inputs (Chemero and Silberstein, 2008). This approach also connects directly with the burgeoning field of network physiology (*cf.* Balagué et al, 2020) in that it treats the human-behavioral system as an integrated network of interconnected organ sub-systems, while considering their complex nonlinear dynamics through specific fitness variables. Further, each fitness variable serves as a proxy for these dynamic interactions within or across sub-systems, depending on the measured characteristic (e.g., neurophysiological, neuromechanical or behavioral), while indexing each across stress magnitudes and over time.

**FIGURE 1:**
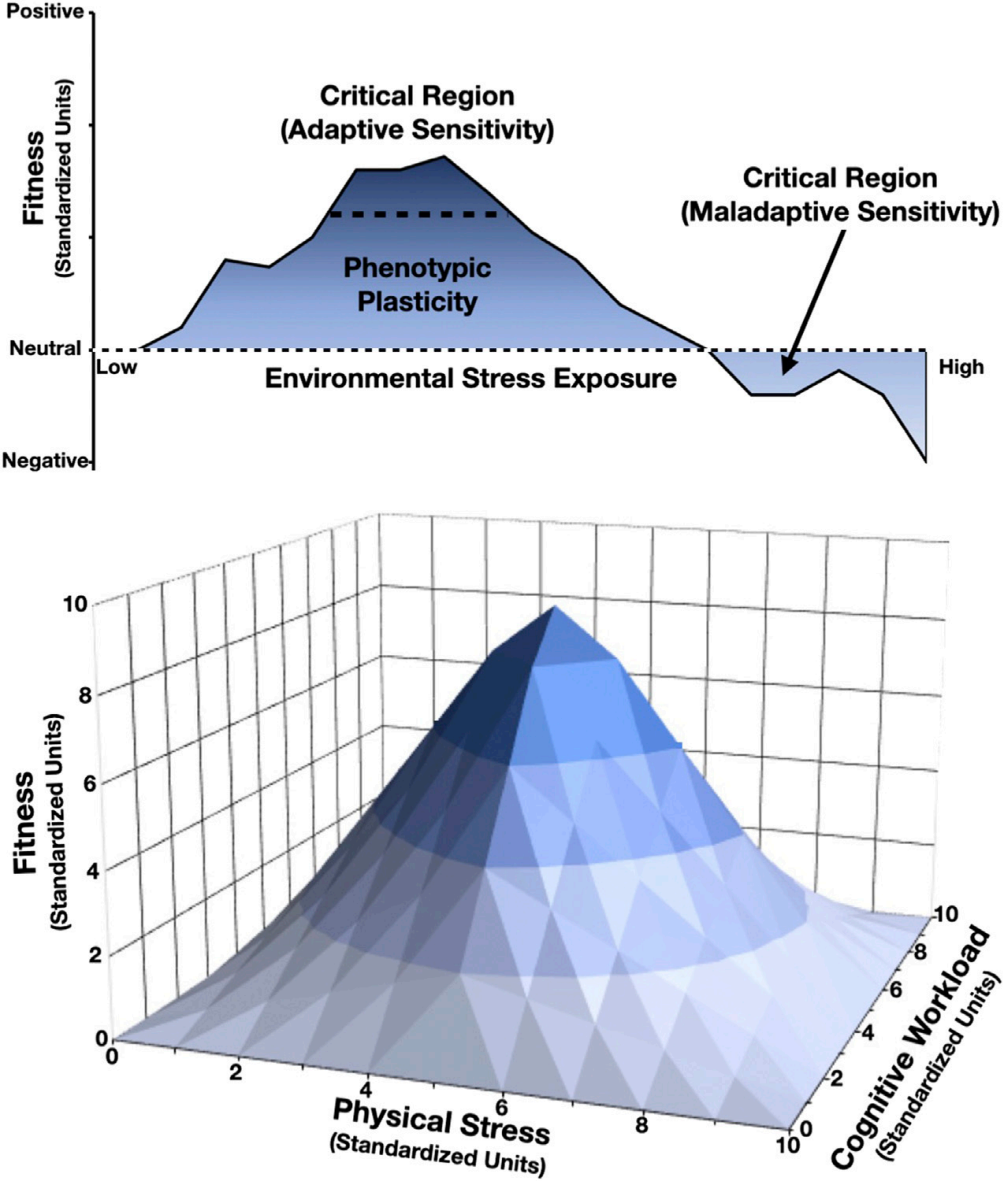
Example phenotypic plasticity profiles across varying levels of stress. *Top*: A 2-dimensional phenotypic fitness curve with fitness (*y*-axis) plotted over environmental stress exposure (*x*-axis). The dotted line at the peak of the curve denotes a critical region of adaptive sensitivity, where the phenotypic fitness is most sensitive to the environmental stress in a positive way. Similarly, the critical region of maladaptive sensitivity is labeled when the phenotypic fitness drops below the baseline, or neutral condition. This is where the phenotypic fitness is most negatively impacted, or overwhelmed, by external stress exposure. The shaded area under (and above) the curve represents one quantifiable index of phenotypic plasticity, or overall adaptability, of the system. *Bottom*: A 3-dimensional phenotypic fitness response surface with phenotypic fitness (*y*-axis) plotted in relation to physical stress (*x*-axis) and cognitive workload (*z*-axis). This provides an example of how additional dimensions of stress or workload can be added to the profile in order to visualize the system’s response to each of these factors.

Essential to this modeling approach is the objective identification of specific stressors and their well-defined magnitudes, to precisely relate phenotypic change as a function of environmental (e.g., performance or daily living) conditions (Kiefer et al., 2018; Kiefer et al., 2022). In doing so, an ordinal stress-fitness response curve can be developed to profile phenotypic expression across a stressor gradient (see Figure 1) and, ultimately, the adaptability of the system *via* the computation of phenotypic plasticity (Agrawal, 2001). *Phenotypic plasticity* is a global fitness characteristic and captures the ability to positively adapt across environmental stressors. It is made up of the interacting components that underlie a given phenotype, and profiles the phenotypic expression across successive measurements of (behavioral) responses relative to increasing stress levels. Phenotypic plasticity therefore accounts for the dynamic nature of adaptive processes underlying functional performance, which emerge from the interaction of the individual with their environment. This metric is likely highly sensitive to the behavioral transitions an individual makes to achieve more efficient performance states and, specifically, behavioral change as a function of environmental change (Kiefer et al., 2018).

A phenotypic plasticity approach leverages several other important principles from evolutionary biology. The first is a connection with dose-response curves (Calabrese and Mattson, 2011) that can inform intervention points and, thus, treatment plans for health or performance enhancement—e.g., the well-defined “inverted-U” (i.e., hormetic) curve of arousal and performance first introduced over a century ago (Yerkes and Dodson, 1908). Foundational to the hormetic response is that a low-dose stress or environmental challenge promotes positive expression (or growth) of the fitness variable under study (Costantini and Borremans, 2019). This type of response profile is ubiquitous in biological systems (Calabrese and Blain, 2011) and provides a profile of a system’s adaptive capability.

**FIGURE 2:**
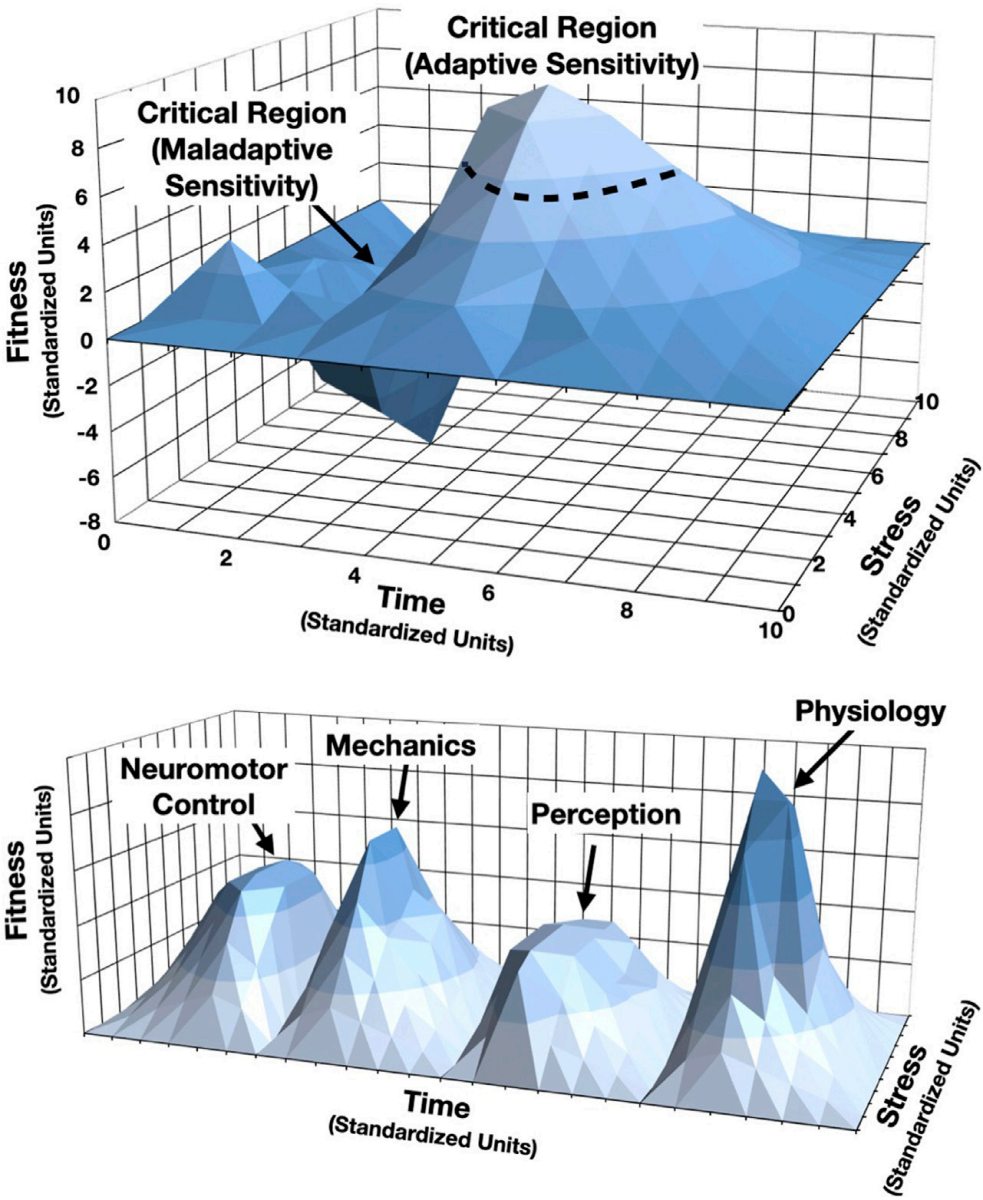
Example 3-dimensional phenotypic plasticity profiles across time. *Top*: A phenotypic fitness surface with a critical window of phenotypic expression as denoted by the arrow indicating the critical region of maladaptive sensitivity of phenotypic fitness in the early stages of skill development (for example) at higher levels of stress. Conversely, the critical region of adaptive sensitivity, denoted by the dotted line, indicates when phenotypic fitness is positively expressed in slightly later stages of skill development, even at these same higher levels of stress. This could be indicative of a match between capability and challenge. *Bottom*: A multi-dimensional phenotypic fitness surface with four phenotypic domains—Neuromotor Control, Mechanics, Perception and Physiology—and their phnoetypic response dynamics across both time and stress. While this is an overly simplified example, in this case, one could see how each dimension of performance can be modeled on the same time/stress scales to allow for phenotypic expression comparison. This approach allows for initial inspection of potential intervention points that are related to critical time windows in the system’s skill development, for example.

A second principle is *critical time windows* (*cf.* Burggren and Mueller, 2015). These have generally been considered relative to crucial periods of biological development and identified as regions when the system is most sensitive and where phenotypic expression is greatest. For the purposes of sport, these can be considered as time windows of enhanced athlete responsiveness. Modeling phenotypic plasticity across a gradient of stress, the third dimensional axis of time can be added. Importantly, while time is an interval variable, the actual units can be based on the temporal ordering of events, with spatial relations relative to important aspects of a training or development cycle (see Figure 2, top). This is a useful profile of individual time windows, but it is likely also possible to model a more generalizable skill development profile averaged across multiple athletes or athletic “genotypes”. One of the most powerful aspects of this approach is the development of multi-domain profiles across both time and stress (*cf.* Burggren and Mueller, 2015). Consider, for example, the varying time scales of physiological, neuromotor, perceptual and skill development processes that take place across a particular season, off-season, or career. By constructing phenotypic profiles across time and stress, one can begin model change among a complex number of interconnected components (see Figure 2, bottom). Note that these are most straightforward when developed as human readable two- or three-dimensional profiles; however, there is no limit to the number of dimensions and scales one can model. Thus, while human readability is paramount, as this approach is further developed it will be critically important to leverage innovative explainable and interpretable machine learning techniques to make sense of high-dimensional networks of components (e.g., Mi et al, 2021), and to detect relevant features of these profiles quickly and accurately for precision training or care.

## 3 Expansion of phenomics to applied sport science

The expansion of phenomics to applied sport science requires profiling key fitness domains associated with athlete performance and health. Although the pre-requisites for success in sport are highly variable, it is common for sport scientists to focus their attention on understanding: 1) physiological and psychological characteristics of the successful competitors, 2) physical, tactical and technical demands of the sport, and 3) interventions that promote desired adaptations. Prior to the development of advanced technology and funding that allowed teams of specialists to focus on athlete support, coaches relied on intuition, experience and often relatively simple measurements of fatigue and performance to guide decision making. Today, many of these general principles remain relevant; however, the methodology involved with evaluating an athlete’s strengths, weaknesses, and readiness to perform can be more sophisticated to yield more meaningful insights. This necessitates innovative technologies that facilitate the high-resolution extraction of phenotypic information with a focus on non-destructive assessments. One such rapidly growing technology is that of computer vision-based extraction of information from video to derive both kinematic outputs, *via* markerless pose estimation (e.g., Uhlrich et al., 2022), and contextualized action recognition (Bertasius et al, 2017; Bertasius et al, 2021) so that unique manifestations of fatigue can be quantified. These tools can be combined with other passive wearable technologies for monitoring biometrics, exercise, and recovery (e.g., global position sensors, accelerometry, heart rate monitoring or even mobile eye tracking). The combination of technologies and methods facilitates integration of biology-based phenomics with a precision human medicine focus. In short, the data can inform the right intervention at the right time, with minimum disruption and maximum efficiency.

The sport of cycling has seen a focus on high precision estimates of body composition including quantification of lower body muscle mass using dual-energy X-ray absorptiometry (DXA; Haakonssen et al, 2016). Sophisticated cycling power meters and cycle ergometers incorporating relevant inertial loads can quantify the maximum power output–exercise duration profiles. During graded exercise tests, heart rate, oxygen update, blood lactate, sweat rates, and perceived exertion can be quantified to document relevant physiological capacities. Wind tunnels can be used to estimate the coefficient of drag area associated with different types of cycling equipment and riding positions. It is also possible to quantify biomechanical manifestations of fatigue during prolonged cycling challenges using force pedals and inverse dynamics (Martin and Brown, 2009). For a given fitness domain, pacing strategies for time trials can be optimized relative to terrain. In addition, new wearable technologies can quantify nutrition, sleep and training loads to provide insights into performance readiness. Recent data examining how muscle adapts to resistance training provides a vivid example of how protein intake prior to sleep can modulate desired adaptations (Trommelen and van Loon, 2016). Sophisticated models that incorporate both stress type and the nutrition available post exercise will be required to predict muscle fitness plasticity. A comprehensive profile of cycling performance could target certain factors and prioritize them for training.

In contrast to an individual sport like cycling where the physiological and biomechanical pre-requisites for success have been identified, team sport introduces additional complexities. For example, professional basketball teams now employ large teams of specialists who focus on player selection, development, competition, and rehabilitation, and use technologies such as computer vision to comprehensively evaluate how players move during practice and games (Monezi et al, 2020). And while support staff tend to focus on enhancing physical, psychological, and technical aptitudes, coaches tend to turn their attention to emergent properties associated with player interactions. Of particular importance in team sport is minimizing risk of injury (Dolan et al., 2022) and building up a culture where players, coaches and staff believe success is inevitable. A phenomics framework can help identify both adaptive and maladaptive regions for fitness variables associated with these factors. Specifically, Pygmalion effects have been described as the performance benefits attributed to preparing in an environment where teachers/coaches portray a strong belief of success in their students/players (Weaver et al, 2015). Unique polling techniques have been shown to be effective at understanding the collective perspective of small groups of individuals and hold promise for evaluating whether teams believe they will improve and ultimately win (Becker et al, 2017). The phenomics-approach in this case can profile these types of psycho-social features and enable the targeting of physical or coaching interventions to enhance plasticity, or adaptation, of specific fitness variables at the right time.

## 4 Considerations and priorities for the future

The phenomics framework is a powerful approach to comprehensively profile and model athlete performance and health. It brings with it important advantages for accurately and efficiently indexing response zones and precisely intervening on relevant fitness variables to enhance an athlete’s adaptability and, ultimately, maximize their peak capabilities. It also scales exceptionally well (*cf.* Agrawal, 2001, for an example in biology). In sport, identified fitness variables can be examined and modeled from an individual athlete up through a team system. For example, a basketball-relevant fitness variable such as contested field goal percentage can be indexed for an individual athlete, a backcourt position group, or for an entire team. Moreover, this could be profiled relative to a specific training session or using player tracking technologies across segments of a season or multiple seasons. It is also possible to look at additional stress variables, such as physical workload during a competitive season, with higher-resolution factors such as shooting performance during more critical game moments or changing defensive capabilities of opponents. Similarly, one could imagine a fitness variable in which the tactical stability, or coherence (e.g., López-Felip et al, 2018), of a team is indexed, and then modeled relative to performance stress.

The phenomics framework also incorporates underlying factors that contribute to phenotypic expression, such as *exposomics*, or an individual’s lifetime environmental exposure history, including lifestyle factors, to better characterize environment-based risk factors (Wild, 2005; Lubelczyk et al., 2013). While this has been predominantly advocated for in human medicine as a scaling up of genomics (Holland, 2017), when considered as additional parameters that act to predispose a phenotype for specific magnitudes of expression relative to environmental stress, exposomics data can integrate well into more complex phenomics-based network models of performance and health. It is important to consider that athletes are exposed to unique environmental factors compared to non-athlete populations, ranging from increased radiation exposure from medical screenings to chemical supplementation and therapeutics (Thevis et al, 2021). Such exposures are easier to track once the athlete is a member of an organization, but each athlete brings with them an exposure history that may dramatically influence several important factors of network physiology and related phenotypic fitness. Therefore, mobile sensors for more accurate and comprehensive evaluation of environmental exposures becomes important to move beyond lower-resolution metrics (e.g., an individual’s geographical home) to higher-resolution monitoring data that can inform exposure and risk models (Loh et al., 2017). In short, all stress responses are not created equal, and exposomics has the potential to further elucidate the complex factors that underlie physiological sub-system interactions that underlie phenotypic expression.

## 5 Conclusion

As the field looks to the future of characterizing and predicting the performance and health of athletes, a phenomics framework will have great utility. It facilitates the modeling of many interacting components and multi-dimensional profiling that is comprehensive and translateable to actionable interventions with high precision. It also promotes personalization through a unit of analysis that is context-driven and accounts for athlete and performance environment factors. To paraphrase famed physiologist Dr. Leon Glass, on the dynamics of biology and health: as scientists and practitioners, we must recognize that advancements may not depend on a breakthrough, but instead often emerge from the “…appropriate use of well-known concepts to vital problems (Glass, 2015, pp. 8).” In the case of phenomics, we must turn to the well-established concepts of fitness, phenotypic plasticity and, ultimately, phenomics, from evolutionary biology as a roadmap for sport science to negotiate the impasse of linear causal models and transform athlete risk management, training, and care.

## Data Availability

The original contributions presented in the study are included in the article/Supplementary Material, further inquiries can be directed to the corresponding author.
